# Psychiatric Manifestations in Fahr's Syndrome: A Case Report

**DOI:** 10.7759/cureus.10770

**Published:** 2020-10-02

**Authors:** Prerak Kumar, Romil Singh, Kaushal Shah

**Affiliations:** 1 Psychiatry and Behavioral Sciences, Lady Hardinge Medical College, Smt Sucheta Kriplani Hospital, New Delhi, IND; 2 Internal Medicine, Metropolitan Hospital, Jaipur, IND; 3 Psychiatry, Griffin Memorial Hospital, Norman, USA

**Keywords:** fahr's syndrome, basal ganglia, calcification, psychosis, paranoia

## Abstract

Fahr's syndrome is a rare neurological entity, primarily impacting basal ganglia with bilateral intracranial calcium deposition. It mainly manifests motor and psychiatric symptoms in affected individuals. After the patient and her family members' consent and proper ethical clearance from the institutional ethical committee, we here report a case presented with a few motor symptoms, features of delirium, and prominent psychiatric symptoms such as disorganized behavior, auditory hallucinations, and delusions. The imaging study found bilateral basal ganglia calcification and edema in the parietal region, primarily on the right side. Laboratory studies revealed mildly low parathyroid hormone and calcium levels, but no significant findings in other investigational tests. Her past medical and psychiatric history were negative, except for her well-adjusted pre-morbid personality. Our aim through this case is to highlight the psychiatric manifestation of a rare neurological syndrome. It also showcases the importance of ruling out medical causes when a patient presents primarily with behavioral symptoms.

## Introduction

Fahr's syndrome is characterized as a rare neurological condition that primarily impacts basal ganglia through its calcification [1]. It mainly involves the lenticular nucleus and the internal globus pallidus [2]. The calcified regions of the brain are determined to have a direct correlation with clinical symptoms [3]. The onset of the disease is typically present in the latter part of life, but reports of its manifestation in childhood and early adulthood are also seen [4]. Neuropsychiatric manifestations are motor symptoms, dementia, amnesia, concentration issues, a wide range of behavioral abnormalities, and psychosis [5]. About 40% of patients with basal ganglia calcification may present with psychiatric symptoms from mania, apathy, or psychosis. The available treatment is mainly symptomatic control, along with treating the underlying causes of calcification. Prognosis is unpredictable, with neurological deterioration being the leading cause of further disability or sometimes death in rare conditions [6].

## Case presentation

A 33-year-old Asian Indian female was brought by her mother to the psychiatric outpatient services. Her medical complaint includes diffuse headache, abnormal behavior, reduced sleep, jerky movements in the left lower limb, decreased appetite, paranoid behavior, and auditory hallucinations. Her paranoid behavior started from the day before her current visit to the hospital and believed that her parents would kill her. She started hearing voices of a man from the last two days and observed occasional jerky movement in the left lower limb from the past fifteen days ago.

**Figure 1:**
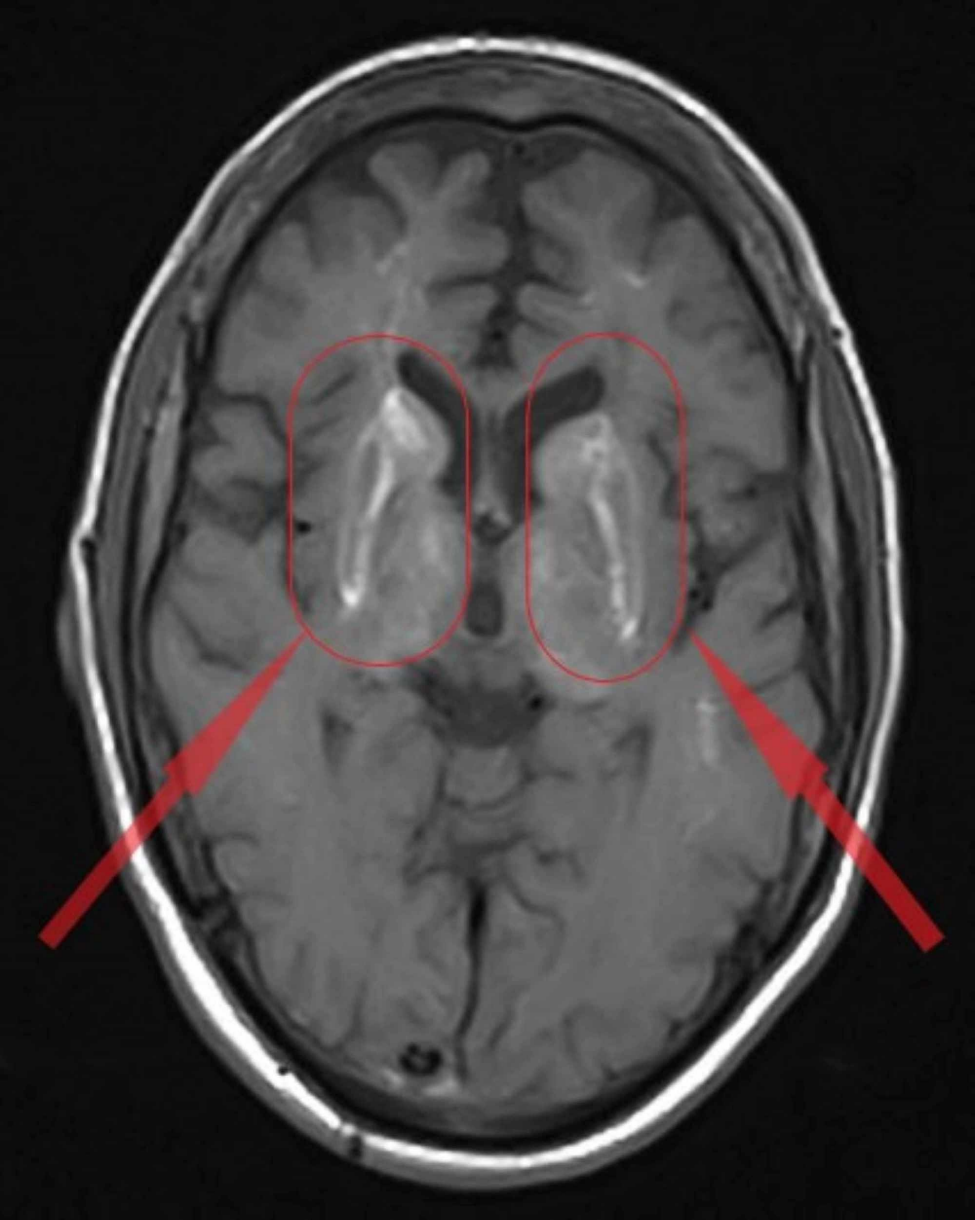
Basal ganglia calcification in the magnetic resonance imaging

The psychiatric symptoms were seen in the patient without any known stressor. The patient was agitated and violent at the time of admissions and later was admitted to the female psychiatry ward. On the first day of the inpatient examination, her general physical examination revealed mild bilateral tremors on outstretched hands with no other signs. Her physical appearance found a lack of grooming and thin built. Upon further evaluation, we observed increased psychomotor activity, irrelevant speech, loosening of association, the delusion of persecution, and auditory hallucinations of the second person. Patient blood investigations showed a mild elevation in the thyroid-stimulating hormone (TSH) value of 5.1 mU/L. Liver function parameters and renal function tests were within normal values. The patient was started with haloperidol 5 mg intramuscularly (IM), promethazine 50 mg IM, and risperidone 2 mg tablet at night time (QHS) on the first inpatient day. On the second inpatient day, she was disoriented to time and person in the noontime and could not identify her mother when interviewed, and her mother corroborated these findings. On the evening of the second inpatient day, her sodium level was 132 mEq/L and chloride at 93 mEq/L. Patient electrolytes abnormalities were rectified by fluid therapy, which resulted in the improvement of her delirium. Her agitation and restlessness were present on the third inpatient day evening with no signs of delirium. Within the next three days in her inpatient care, the patient's agitation and hallucination increased even after tab risperidone was optimized up to 6 mg sequentially. On the sixth inpatient day, magnetic resonance imaging (MRI) of the brain reported bilateral basal ganglia calcifications mainly in the caudate nucleus, as seen in Figure 1. 

A neurologist was consulted on the seventh day of her admission due to her complaint of continuous headache and occasional mild jerky movements. On examination, tremors on outstretched hands were confirmed with poor cortical sensation in the sensory examination. Oral naproxen 500 mg tablet once a day (OD) and propranolol 40 mg tablet OD was initiated for headaches and tremors, respectively. The patient was found febrile on her ninth day of admission, and acetaminophen 500 mg was started immediately on a pro re nata (PRN) basis. Her fever subsided, but her tremors, agitation, auditory-hallucinations persisted. 

Laboratory workup related to the endocrinology consisted of calcium, parathyroid (PTH), prolactin, and serum vitamin B12 assays. Her PTH level was found to be mildly decreased (actual result: 8.9 pg/mL; normal range: 10-65 pg/mL), and serum calcium was also slightly decreased (actual result: 7.4 mg/dL; normal range: 9-10.5 mg/dL) with rest parameters normal. Based on these findings, levothyroxine 25 μg and calcium 500 mg OD dose was initiated. On the eleventh day, her father visited the ward and reported that she had a past history of two episodes of seizures, which occurred about a month ago. On the same day, based on her MRI, the radiologist found findings suggestive of Fahr's syndrome, and also, a neurologist was consulted for her seizure episode. Sodium valproate 500 mg twice a day (BID), folic acid tablet 5 mg OD, propranolol increased to 60 mg OD from initial 40 mg for persisting tremors. Risperidone was optimized to 8 mg with lorazepam 2 mg night dose (QHS) was started. In the next few days, around the fifteenth day of her admission, the patient auditory hallucinations were still persisting, but her agitation, delusions, and paranoia for family members were reduced as reported by parents. The patient was discharged due to clinical improvement on the seventeenth day of admission on the above treatment with two weeks follow up from psychiatry, endocrinology, and neurology departments. The patient's auditory hallucinations were still present on the follow-up visit with rest symptoms of delusion, agitation, tremors improving as reported by parents. Tablet amisulpride 100 mg and tablet trihexyphenidyl 1 mg was augmented in the above regime and was scheduled to follow up after a month. After one month, the patient did not report, and family members informed through telepsychiatry consultation that the patient reported only 10% to 15% improvements in her hallucinations and headache; her agitation, delusion, tremors subsided as reported. 

## Discussion

A study by Ramis et al. determined the disturbance in the calcium and phosphate metabolism in this rare neurological disorder, which leads to hypocalcemia and hypoparathyroidism [7,8]. Other possible etiologies of Fahr's syndrome include infections, metabolic, and genetic diseases [9]. A research study reported 0.3% to 1.3% of neurological findings due to basal ganglia calcifications, which supports the clinical symptoms of the patient with laboratory and imaging studies [10,11]. As seen in this case, general clinical features are headache, tremors, motor symptoms, psychosis, mania, seizures, and memory changes [12]. Another case of Fahr's syndrome presented with motor symptoms, syncope, delirium, and hypocalcemia with few psychiatric issues. However, our case primarily showed features of delirium and psychotic features in the form of disorganized behavior, pacing around, and irritability. Early-onset psychotic presentation in Fahr's syndrome is generally presented with a mean age of 30.7 years with limited movement disorder involvement and late-onset with a mean age of 49.4 years found to be associated with dementia, amnestic changes, and movement disorders. Fahr's syndrome treatment should focus on treating underlying causes; however, the response rate to medications in management varies. Psychiatric symptoms are reported to higher variability of response rate in management [13]. With thorough diagnostic approach and treatment, Fahr's syndrome symptoms can be managed appropriately, especially the behavioral and psychiatric issues.

## Conclusions

In summary, psychiatrists must consider neurological or medical causes for any psychiatric manifestations and cautiously rule out behavioral problems in suspected cases through meticulous investigation. This case highlights rare syndrome as an underlying cause of psychosis associated with motor abnormalities. It also provides an insight into the vital role of neuroimaging and endocrinological hormone assays for revealing the etiology of psychosis. Symptomatic management of clinical signs has shown its significance in controlling behavioral and motor symptoms. This case aims to educate and enlighten clinicians about the rare neurological syndrome that requires thorough medical and neurological evaluation for correctly diagnosing its primary psychiatric illness.
